# Correction: Drug targeting CYP2E1 for the treatment of early-stage alcoholic steatohepatitis

**DOI:** 10.1371/journal.pone.0247690

**Published:** 2021-02-19

**Authors:** The PLOS ONE Staff

There are errors in the author affiliations. The correct affiliations are as follows:

Torsten Diesinger^1, 2, 3^*, Vyacheslav Buko^4,5^, Alfred Lautwein^1^, Radovan Dvorsky^6,7^, Elena Belonovskaya^4^, Oksana Lukivskaya^4^, Elena Naruta^4^, Siarhei Kirko^4^, Viktor Andreev^8^, Dominik Buckert^1,9^, Sebastian Bergler^1^, Christian Renz^1^, Edith Schneider^10^, Florian Kuchenbauer^10,11^, Mukesh Kumar^12^, Cagatay Günes^12^, Berthold Büchele^13^, Thomas Simmet^13^, Dieter Müller-Enoch^1^, Thomas Wirth^1^, Thomas Haehner^1^

**1** Institute of Physiological Chemistry, Ulm University, Ulm, Germany, **2** Department of Internal Medicine, Neu-Ulm Hospital, Neu-Ulm, Germany, **3** Chair of Biochemistry and Molecular Medicine, Witten/Herdecke University, Witten, Germany, **4** Division of Biochemical Pharmacology, Institute of Biochemistry of Biologically Active Substances, Grodno, Belarus, **5** Department of Biotechnology, University of Medical Sciences, Bialystok, Poland, **6** Institute of Biochemistry and Molecular Biology II, Medizinische Fakultät der Heinrich-Heine-Universität Düsseldorf, Düsseldorf, Germany, **7** Max Planck Institute of Molecular Physiology, Dortmund, Germany, **8** Department of Medical Biology and Genetics, Grodno State Medical University, Grodno, Belarus, **9** Department of Internal Medicine II, University Hospital Ulm, Ulm, Germany, **10** Department of Internal Medicine III, University Hospital Ulm, Ulm, Germany, **11** University of British Columbia, Terry Fox Laboratory, Vancouver, Canada, **12** Department of Urology, University Hospital Ulm, Ulm, Germany, **13** Institute of Pharmacology of Natural Products and Clinical Pharmacology, University Ulm, Ulm, Germany

The publisher apologizes for the error.
