# Supplementary figures and images for: Drug targeting CYP2E1 for the treatment of early-stage alcoholic steatohepatitis
Source: PLoS One. 2020 Jul 23;15(7):e0235990. doi: 10.1371/journal.pone.0235990 (PMC7377376; doi:10.1371/journal.pone.0235990)

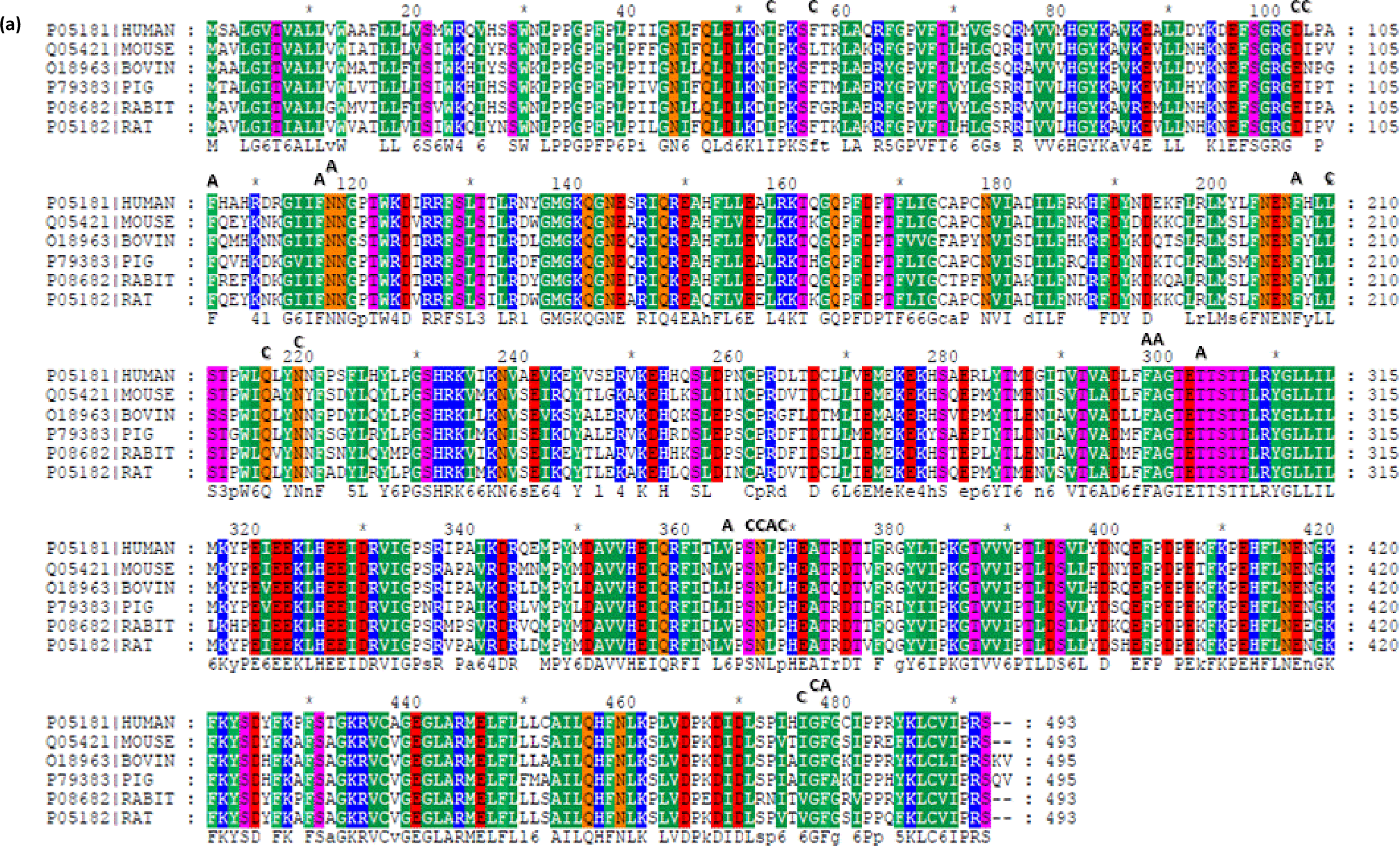

Supplement: S1 Fig — These sequences originate from the species human, mouse, cow, pig, rabbit and rat. The sequence homology is compared with structural components of the active site A and the substrate access channel C. Colors encode the following: green (hydrophobic residues), red (acid), blue (lysine, arginine, histidine), orange (asparagine, glutamine), pink (serine, threonine). (TIF) [file pone.0235990.s001.tif]

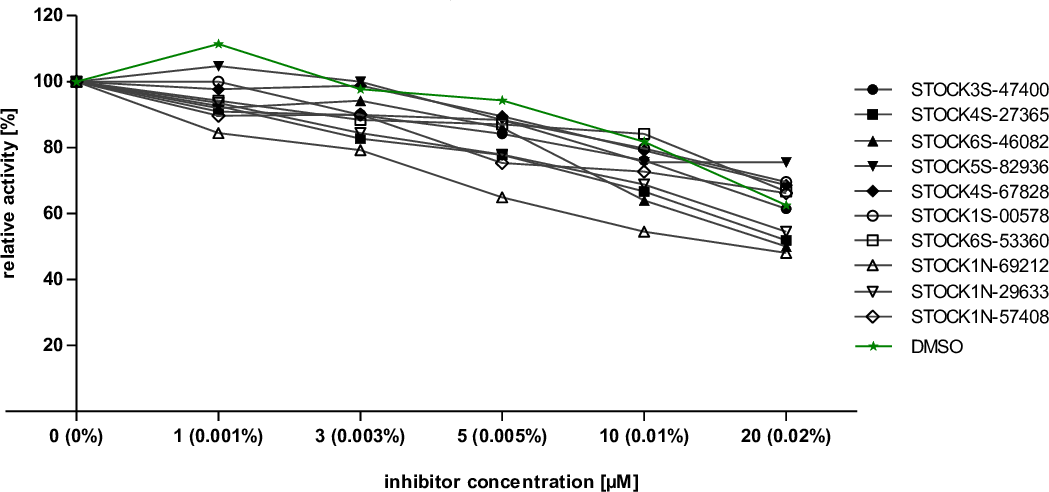

Supplement: S2 Fig — To ascertain whether an imidazole containing compound is already commercially available and better suited for development as a drug can-didate, we performed a large virtual library screening of the ZINK database. We found 14,738 hits. The 5,261 commercially available compounds could be classified as 4,710 synthetic and 551 natural molecules. After docking and final scoring process, the seven best synthetic and the three best natural candidates were purchased and tested in vitro for their potential to inhibit CYP2E1 activity. Different concentrations of these compounds dissolved in DMSO were added to 110 μM p-Nitrophenol in 100 mM HEPES buffer pH 7.6 and SUPERSOMES™ with 50 nM human CYP 2E1 to calculate relative activity values. All ten compounds showed an impres-sively weaker inhibitory effect than I-ol, whereby the natural compound STOCK1N-69212 showed the strongest inhibitory effect among all other library compounds with an activity rate of 48.1% at a final concentration of 20 μM. The real inhibition potential of STOCK1N-69212 must be weaker because the solvent DMSO has an IC50 value of 0.065% (v/v) (doi: 10.6084/m9.figshare.12387107, https://figshare.com/s/f3ec912565aee777ea06), i. e. 20 μM corresponds to a DMSO volume of 0.02% (v/v). (TIF) [file pone.0235990.s002.tif]

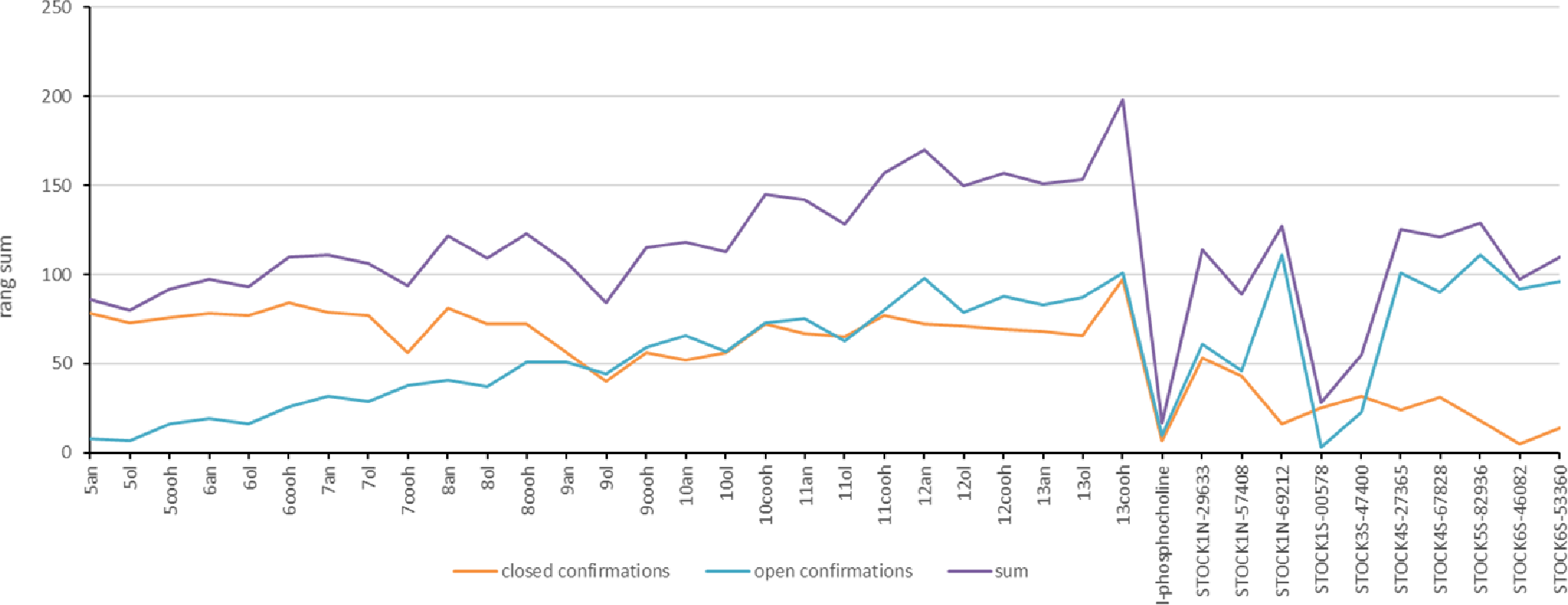

Supplement: S3 Fig — We calculated the median of predicted affinities of the best poses for each ex-perimental substance binding to all six protein structures as provided by default output. After calculating the rank for each conformation, the rank sum for each compound was calculated for the ‘open protein conformations’, the ‘closed protein conformations’ and both conformation types. Although there were no major conformational changes in the protein backbone, ligands occupied a versatile binding site, whose shape is determined by the rotameric states of Phe 298 and Phe 478. In crystal structures with imidazolyl-octanoic acid, -decanoic acid and -dodecanoic acid as stabilizing ligands, the alkyl chain occupied a hydrophobic channel of protein helices which was exposed by a rotation of the Phe 298 side chain. This led to a switch of the ‘closed protein conformation’ to the ‘open protein conformation’. For the ‘closed protein conformations’, the alkyl derivatives showed a nearly constant binding affinity irrespective of their chain length, while the library compounds almost failed. On the other hand, there was a clear influence of the alkyl chain length on affinity to the ‘open protein conformations’, with the functional group -COOH increasing the affinity with 13cooh as the best scored compound. I-phosphocholine with its bulky head group was one of the worst binding compounds independent of protein conformation. (TIF) [file pone.0235990.s003.tif]

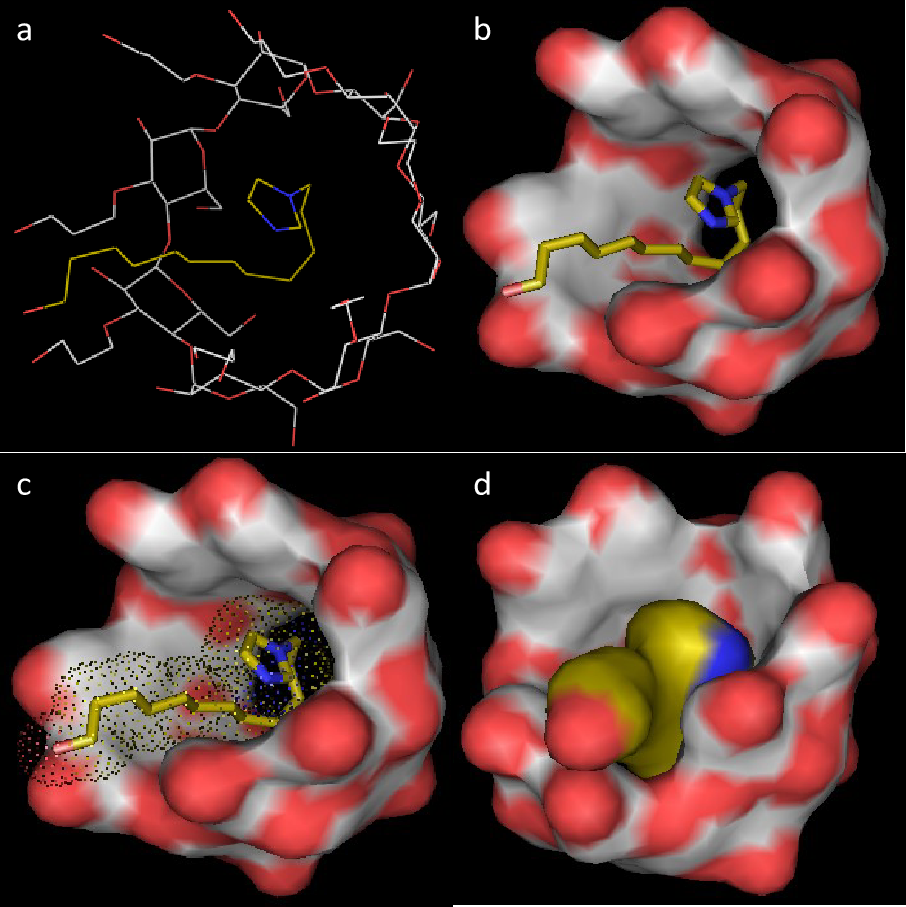

Supplement: S4 Fig — (a) Wire Frame Mode of both molecules. (b) Stick Mode of I-ol and Surface Mode of HPβCD. (c)+(d) Surface Mode of both molecules; carbon of HPβCD (white), car-bon of I-ol (yellow), oxygen (red), nitrogen (blue). The illustrated conformation represents one of the most thermodynamically stable conformations of both in-teraction partners. The interior of the structure encloses a hydrophobic cavity of 260 nm diameter. The hydrophobic part of I-ol fits entirely into this cavity due to its flexible structure. The hydrophilic outer surface guarantees solubility in aque-ous environments (buffer systems, cell culture medium and blood). The hydroxyl group of I-ol generates polar interactions with hydroxyl groups on the outer sur-face of HPβCD (more precisely: a hydrogen bond, which is not shown here). This bridge gives additional stability to the formed complex formed, which leads to a higher solubility of I-ol. (TIF) [file pone.0235990.s004.tif]

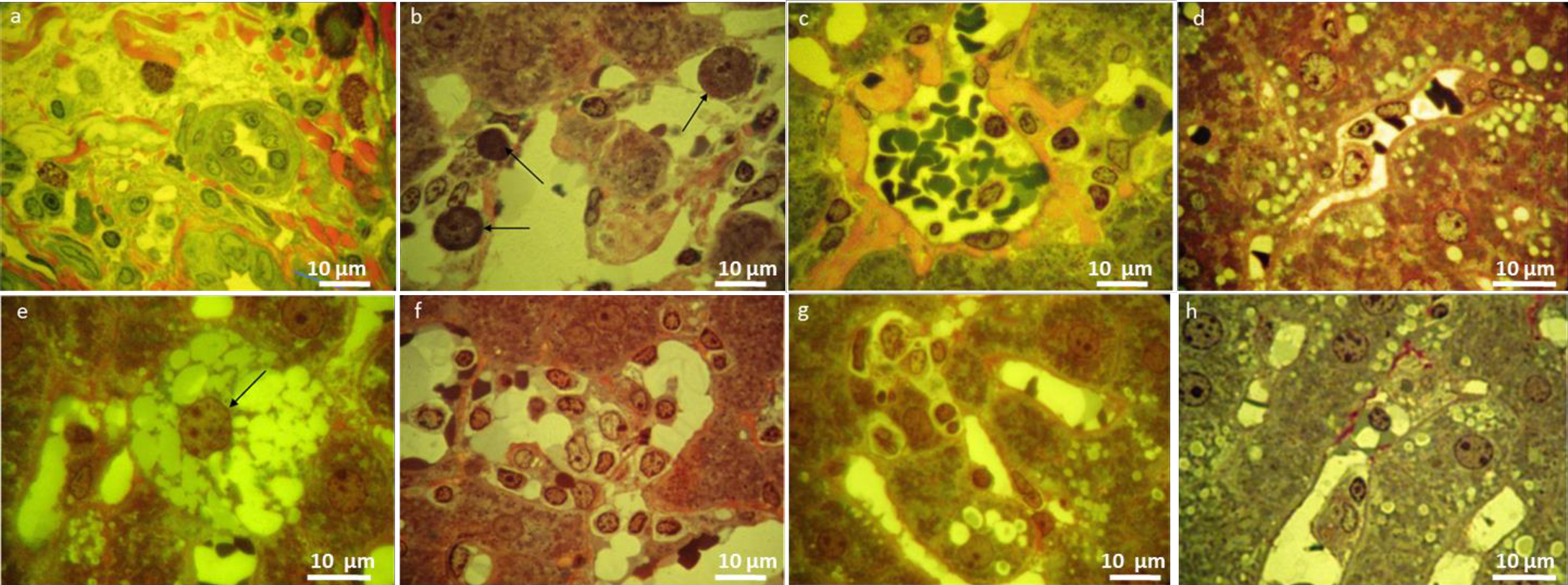

Supplement: S5 Fig — Ten sections were made from each liver—with six or eight animals per group. The magnification of the objective was 100-fold and that of the ocular 10-fold. The following are representative illustrations of these liver sections: (a) Portal tract of a healthy animal (b)-(g) Ethanol treated group (b) Necrosis near central vein (arrows point to apoptotic hepatocytes) (c) Massive layer of connective tissue (pink color) around the central vein lined with endothelium. (d) Microvesicular hepatocyte infiltration. Macrophages with large nucleoli (sign of intensive protein synthesis) that migrate into sinus. (e) Macrovesicular fatty dystrophy. The cell nucleus (arrow) is surrounded by massive lipid droplets (f) Enlargement of the sinusoidal lumen by destruction of the hepatocytes. Cluster of inflammatory leucocytes in the lumen. (g) Destruction of hepatocytes with cytotoxic lymphocytes. (h) Characteristic picture with significantly reduced destruction of liver tissue in the periportal area, microvesicular fatty infiltration and individual small lymphocyte aggregates in sinusoids by administration of I-ol (4 mg/kg b.w.). Comparable results were obtained with I-ol (40 mg/kg b.w) with only single small aggregates in sinusoids, neither apoptotic cells nor infiltrations of the portal and perivenular area with macarophages or lymphocytes. Each scale bar indicates 100 μm. (TIF) [file pone.0235990.s005.tif]

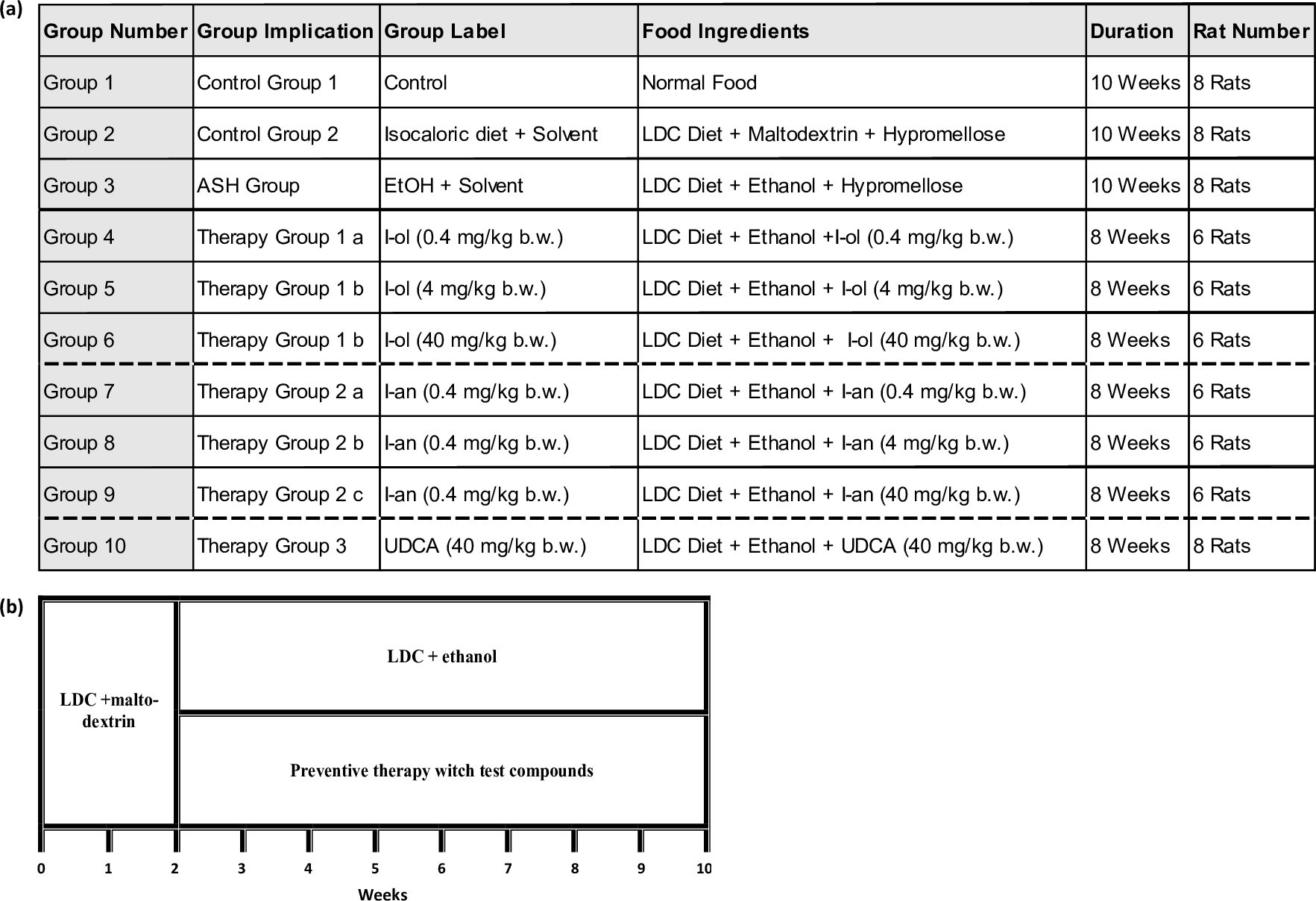

Supplement: S1 Table — Scheme of animal studies (a) Group differences: Rats were divided into two control, one disease group and seven treatment groups. The disease group and the treatment groups received a diet with 5% (m/m) ethanol. (b) Time frame of the experiment: Ethanol feeding was introduced gradually over an adaption period of 2 weeks prior to the start of the experiment. The tested compounds and Hypromellose were administered daily from the beginning of ethanol feeding until the end of the experiment. (TIF) [file pone.0235990.s006.tif]

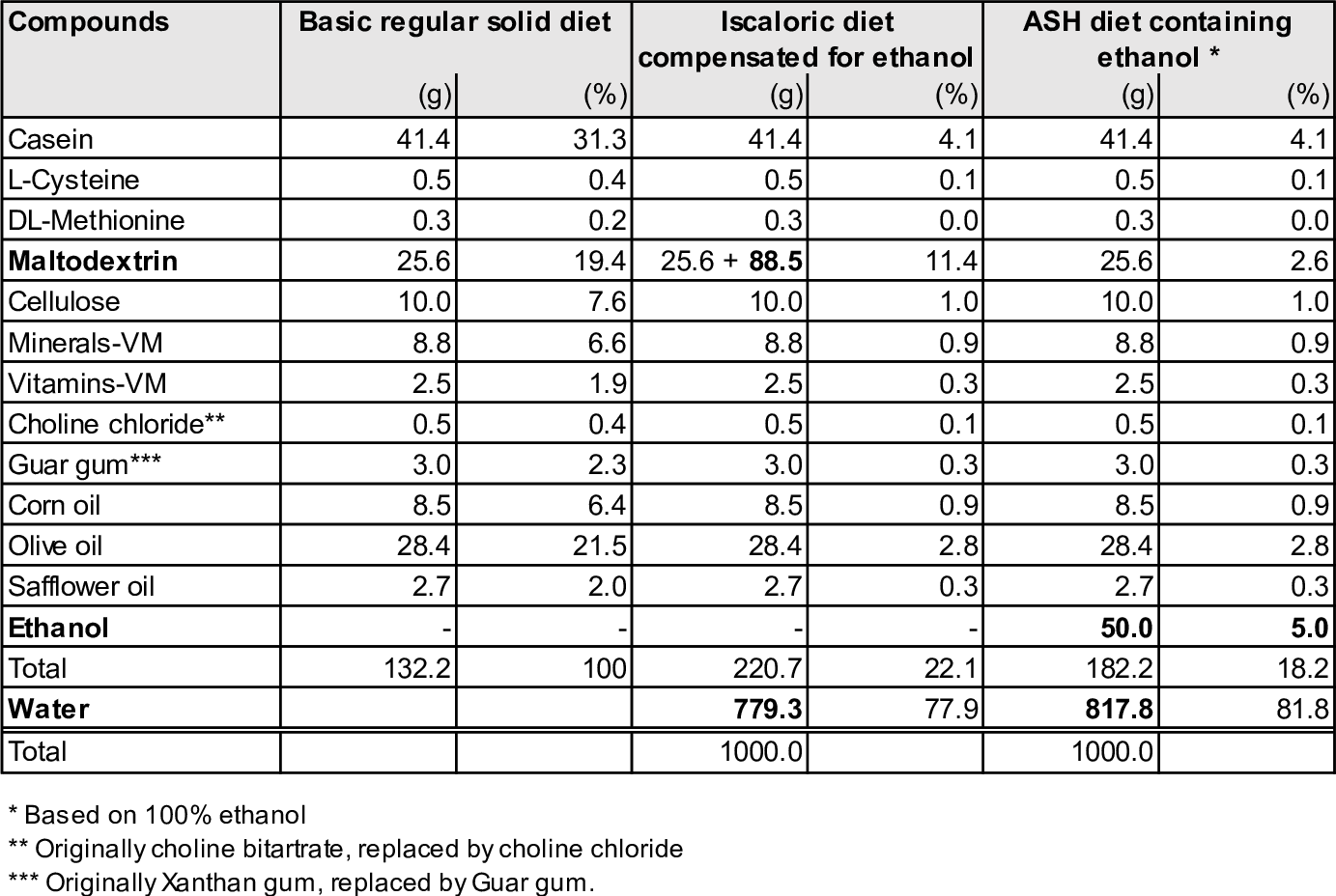

Supplement: S2 Table — (TIF) [file pone.0235990.s007.tif]

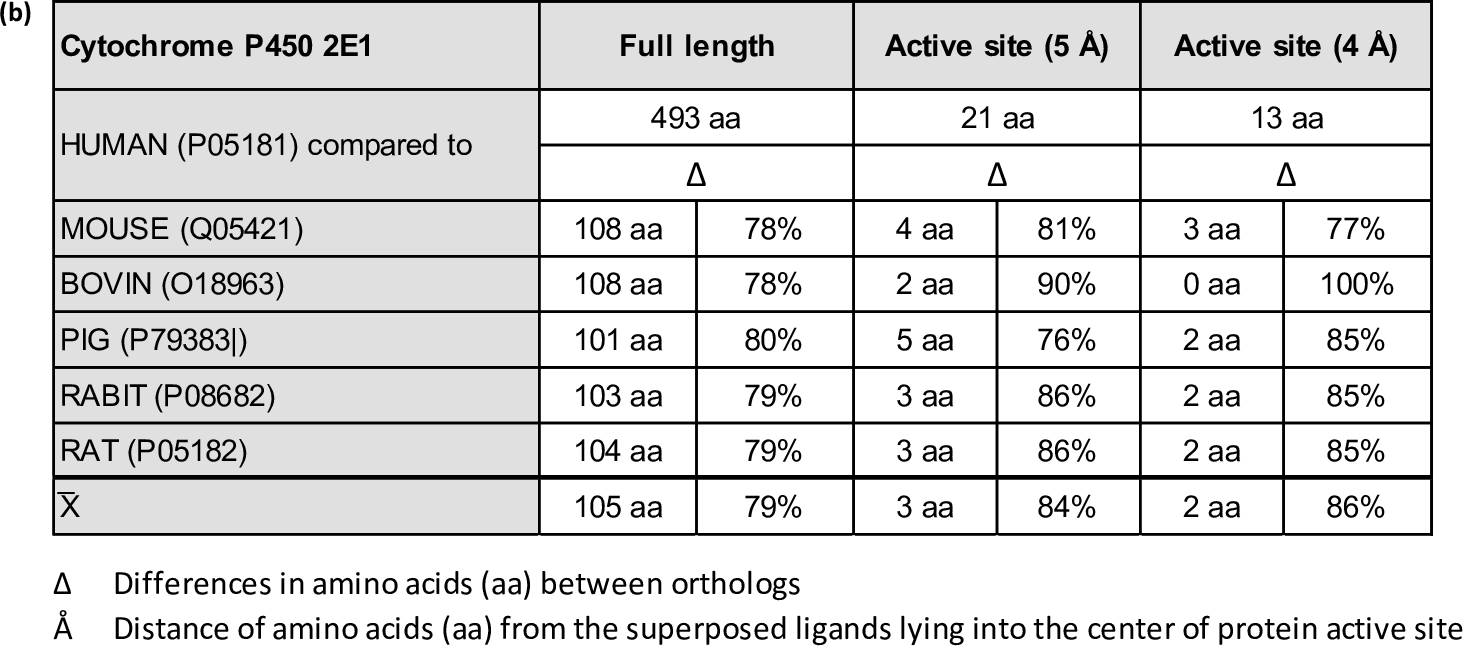

Supplement: S3 Table — (TIF) [file pone.0235990.s008.tif]

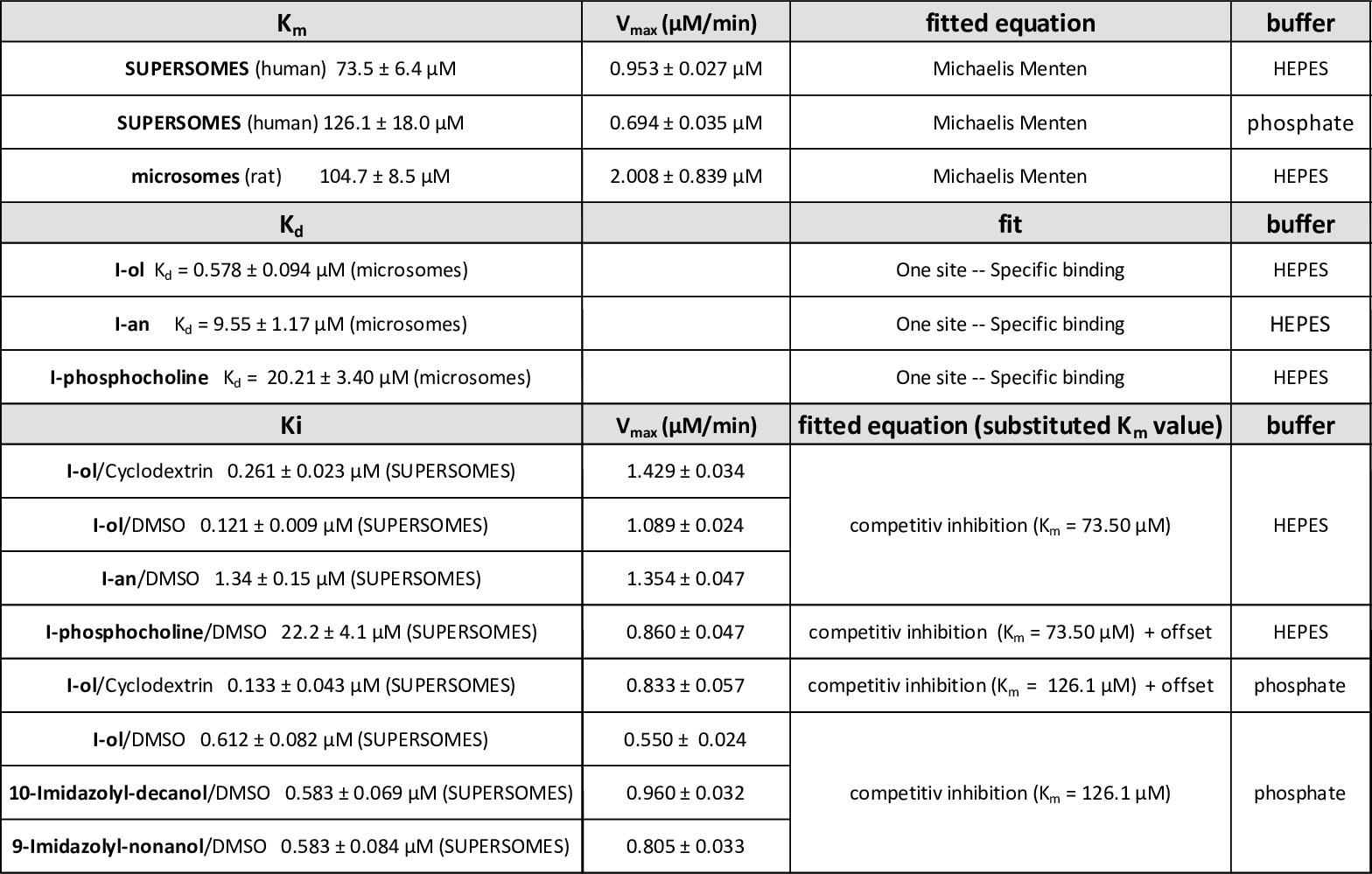

Supplement: S4 Table — Nonlinear fitting to calculate Ki values was achieved using competitive inhibition as model including an offset in the case of I-phophocholine. Kd values were calculated by nonlinear fitting of the experimental data to an equation including one specific binding site. The calculated apparent inhibition constant diminished with increasing ionic strength (i.e. HEPES to phosphate buffer) and depends on the way I-ol is solubilized (i. e. DMSO or cyclodextrin) as well. The Kd value of 578 nM obtained from a titration of rat liver microsomes with I-ol is close to the catalytic inhibition constant obtained with SUPERSOMES™. (TIF) [file pone.0235990.s009.tif]

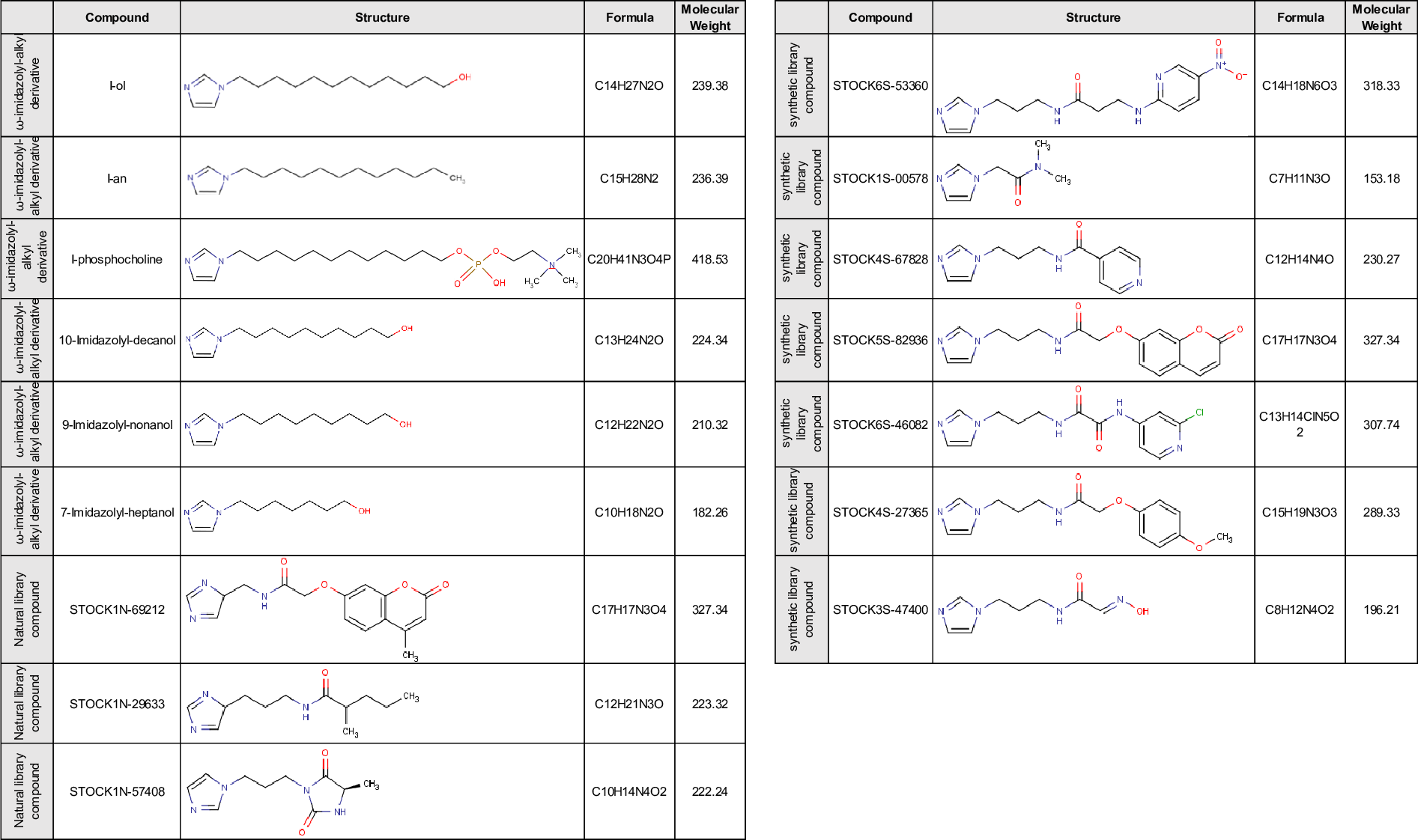

Supplement: S5 Table — (TIF) [file pone.0235990.s010.tif]

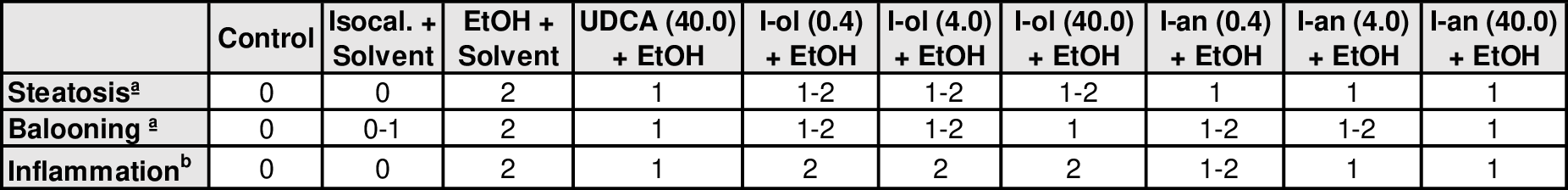

Supplement: S6 Table — ªSteatosis and ballooning: 0, none; 1, ≤ 25%; 2, 26–50%; 3, ≥ 51–75% of liver parenchyma. bInflammation: 0, none; 1, < 5 signs of inflammation; 2, > 5 signs of inflammation in the microscopic field at a 40x magnification. (TIF) [file pone.0235990.s011.tif]

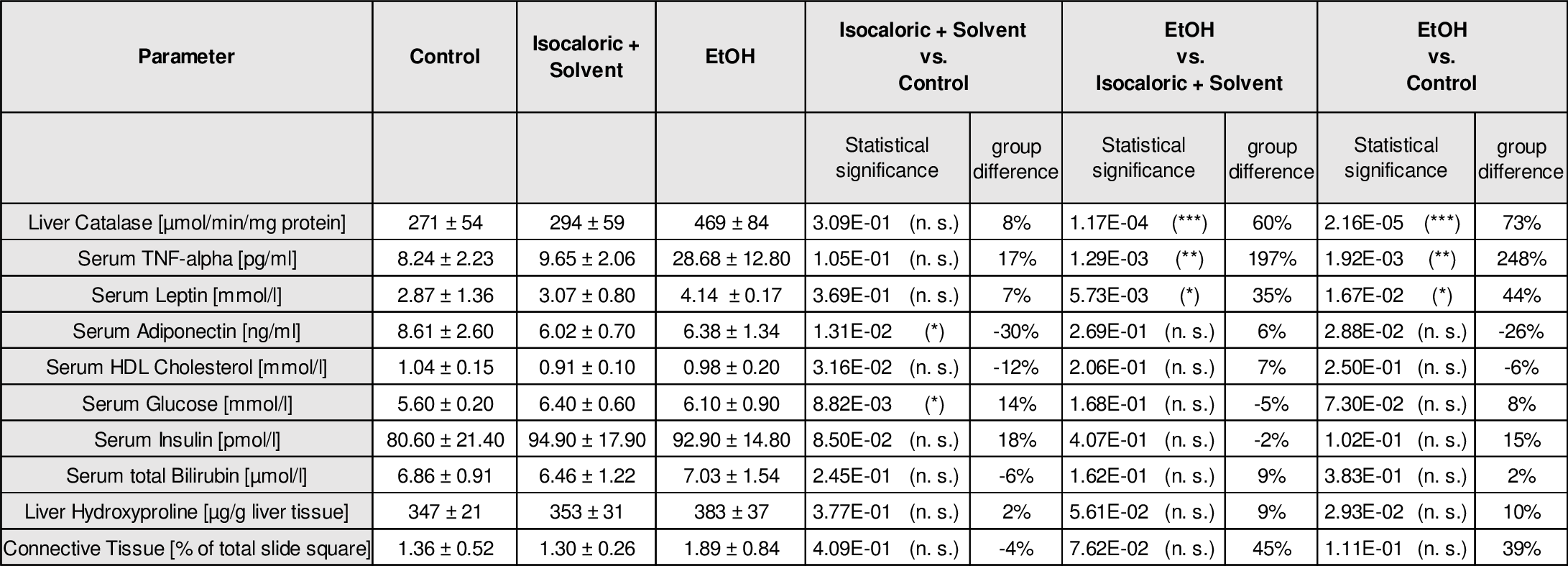

Supplement: S7 Table — (TIF) [file pone.0235990.s012.tif]
