# Supplementary material for: Drug targeting CYP2E1 for the treatment of early-stage alcoholic steatohepatitis
Source: PLoS One. 2020 Jul 23;15(7):e0235990. doi: 10.1371/journal.pone.0235990 (PMC7377376; doi:10.1371/journal.pone.0235990)
Supplement: Attachment — Submitted filename: Response to Reviewers.docx [file pone.0235990.s016.docx]

**General revision**

1) *Thank you for your ethics statement: 'The animal study was conducted at the Institute of Biochemistry of Biologically Active Substances, Department of Biochemical Pharmacology. This is a member of the National Academy of Sciences of Belarus. The responsible ethics committee approved this study and follow-up studies. According to the disclosed protocol in the submitted manuscript, the rats were anesthetized by intraperitoneal injection of a five percent phenobarbital solution and then sacrificed by cutting the abdominal aorta.'*
   a) *Please amend your current ethics statement to include the full name of the ethics committee/institutional review board(s) that approved your specific study.*

The care, use, and procedures performed on these rats were approved by the Ethic Committee of the Institute of Biochemistry of Biologically Active Compounds, National Academy of Sciences, Belarus.

b) *Once you have amended this/these statement(s) in the Methods section of the manuscript, please add the same text to the “Ethics Statement” field of the submission form (via “Edit Submission”).*

Please refer to the inserted statement in section *“Ethics Statement” field of the submission form*

2) *Please provide additional information about each of the cell lines used in this work, including any quality control testing procedures (authentication, characterisation, and mycoplasma testing). For more information, please see* [*http://journals.plos.org/plosone/s/submission-guidelines#loc-cell-lines*](http://journals.plos.org/plosone/s/submission-guidelines#loc-cell-lines)*.*

- HepG2 cells:

The cells were purchased from ATCC in 2012 and have since been used in various research projects within the department according to the recommended culture methods.

Knowles BB, Aden DP. Human hepatoma derived cell line, process for preparation thereof, and uses therefore. US Patent 4,393,133 dated Jul 12 1983

ATCC® HB-8065™

- E47 and C34 cells:

Both cell lines were kindly provided to us by Prof. Dr. Cederbaum (Icahn School of Medicine at Mount Sinai, NY) for research purposes in 2012. The proper handling of the cell lines was the same as for the HepG2 cells.

Chen Q, Cederbaum AI. Cytotoxicity and Apoptosis Produced by Cytochrome P450 2E1 in Hep G2 Cells. Molecular Pharmacology. 1998;53(4):638-48.

Mari M, Cederbaum AI. CYP2E1 overexpression in HepG2 cells induces glutathione synthesis by transcriptional activation of gamma-glutamylcysteine synthetase. J Biol Chem. 2000;275(20):15563-71

- OCI-AML3, NB4, MOLM-13 and MOLP-8 cells: All five cell lines were kindly provided to us by Dr. Dr. Florian Kuchenbauer (University of British Columbia, Terry Fox Laboratory, Canada) for research purposes in 2012. There were owned by the University Hospital Ulm, Clinic for Internal Medicine III - Haematology, Oncology, Rheumatology, Infectious Disease and were part of the common portfolio of cell lines under investigation.

3) *We noted that several of your references did not auto populate and instead the manuscript contains the following "Error! Reference source not found", please replace this with the appropriate reference during your next revision."*

The reason for this error message was missing links of the supplement figures and tables in the text. These are now fixed.

4) *We note that you have included the phrase “data not shown” in your manuscript. Unfortunately, this does not meet our data sharing requirements. PLOS does not permit references to inaccessible data. We require that authors provide all relevant data within the paper, Supporting Information files, or in an acceptable, public repository. Please add a citation to support this phrase or upload the data that corresponds with these findings to a stable repository (such as Figshare or Dryad) and provide and URLs, DOIs, or accession numbers that may be used to access these data. Or, if the data are not a core part of the research being presented in your study, we ask that you remove the phrase that refers to these data.*

- Pages 16+32: "It was not possible to calculate a reliable kinetic parameter, since the necessary concentrations of the solvent DMSO have an inhibitory effect on CYP2E1 (Data not shown)."

🡪 doi: 10.6084/m9.figshare.12387107, <https://figshare.com/s/f3ec912565aee777ea06>

- Page 17: "The activity in the cell types AML and myeloma MOLP-8 was below the detection limit of the assay (data not shown), while ROS production could be detected (Fig 2c), probably by other means."

🡪 The term "data not shown" was omitted. The FACS method used was not sensitive enough to generate usable data in AML and myeloma cell lines, so that a graphical representation was not possible.

5) *At this time, we request that you please report additional details in your Methods section regarding animal care, as per our editorial guidelines:*
   a) *Please state the source and number of mice used in the study*

A total of 68 female Wistar rats (Institute of Pharmacology and Biochemistry Breeding House, Minsk, Belarus) were used in the study.

b) *Please provide details of animal welfare (e.g., shelter, food, water, environmental enrichment)*

Each rat group (n=6-8) was housed in a separate cage and had free access to the liquid diet during the full study period. Prior to the study, the animals were habituated for seven days to the cage conditions and experimental handling. The rats were maintained in a controlled experimental environment (20–25°C, 40–50% humidity, 12 h light per day, room with a 12-h light/dark cycle).

c) *Please describe the post-operative care received by the animals, including the frequency of monitoring and the criteria used to assess animal health and well-being.*

In our experimental design the animals were not subjected to surgery.

6) *At this time, we ask that you please provide scale bars on the microscopy images presented in Figure 6 and refer to the scale bar in the corresponding Figure legend.*

On the microscope images shown in Figure 6 and S6 Figure, the scale bars have been inserted according to your comment.

**Reviewer 1**

7) *"Figure2:* *In case there is no reference standard available, it might allow easier comparison if the values are normalized to control HepG2 instead of plotting Fluorescence Intensity units, as those might differ between equipment/ lab."*

The reference of the investigated cell lines to HepG2 cells leads to a normalization, but this does not allow a correct representation of the effect of I-ol. The reason for this is that the ratio of the mean values between the individual cell lines and HepG2 on the one hand and between the control and therapy group on the other hand sometimes differ considerably or even behave inversely to each other.

**Table 1:** **Normalization of the value to HepG2 cells**

As a result, the existing therapeutic effect of I-ol in the E47 cell line can no longer be represented, which is shown in the following figure.

**Figure 1: ROS production in different cell lines after normalization**

In addition, the aim of this experiment was to test whether ROS production in the genetically unaltered HepG2 cell line is statistically significantly influenced by I-ol. This cell line should not be used as an internal standard before the start of the experiment. A retrospective change in the experimental design would have an undesired effect on the statistical evaluation.

Even if the measured fluorescence values show a dependence on the instrument used and laboratory conditions, the effects shown should be reproducible.

## *"Please add corresponding data for I-an and UDCA in cell lines."*

I-ol showed the strongest effect on the activity of CYP2E1 in both in silico calculations and inhibition studies. Considering the literature on the effect of ω-imidazolyl alkanoic acids and other alkane derivatives, this was not surprising. Several independent studies have shown that 12-(imidazolyl)-dodecanoic acid, the next higher oxidation state of I-ol, is the most potent inhibitor of CYP2E1 and CYP4A1/11 among ω-imidazolyl alkanoic acids and other alkane derivatives (Alterman, Chaurasia et al. 1995; Bambal and Hanzlik 1996; Bambal and Hanzlik 1996; Lu, Alterman et al. 1997; Porubsky, Battaile et al. 2010). The publication "Human Cytochrome P450 2E1 structures with fatty acid analogues reveal a previously unobserved binding mode" (Porubsky, Battaile et al. 2010) is the first scientific paper to answer the question of how the crystal structure of CYP2E1 is calculated as a function of medium-length fatty acids. The result is that 12 (imidazolyl) dodecanoic acids binds with the highest affinity to CYP2E1 compared to other ω imidazolyl alkanoic acids.

UDCA was only intended as a reference substance for animal studies. A significant inhibitory interaction with CYP2E1 has not been described in the literature so far and was not expected based on the molecular structure. Therefore, we deliberately omitted a cellular inhibition study with UDCA. The main question was whether the most potent inhibitor works not only in the subcellular system but also in the cellular system.

In parallel, we performed an acute toxicity study with the compounds I-ol, I-an and I-phosphocholines, which showed a clear superiority of I-ol as the best tolerated compound. The results of this study can be reviewed in the article "ω-imidazolyl-alkyl derivatives as new preclinical drug candidates for NASH therapy", also submitted to PLOS ONE. The planned cell experiments could only be carried out after a galenics study had been successfully completed. Since this already was a scientific challenge for one drug candidate, we decided to further develop I-ol.

## *"Did you perform Pharmacokinetic studies in vivo?"*

We conducted three proof-of-concept studies, which are currently being reviewed by PLOS ONE.

Following these studies, we performed a single pharmacokinetic study with I-ol to get evidence for a possible further development of this lead compound. The complete study is summarized in the Word document " First pharmacokinetic study of I-ol".

In summary, there is a large discrepancy between the proven biological effect of I-ol and the very low bioavailability of this substance. Subsequent studies could clarify the underlying causality.

## *"As reference for the reader, how does the drug concentration in cell culture correlate to serum/ in vivo concentrations?"*

Assuming a maximum oral bioavailability of 0.1 percent for I-ol, the following comparison results:

The peroral intake of 0.4 mg I-ol/kg body weight of the rat leads to a blood concentration of 6.3 ng I-ol/ml, which corresponds to 26.3 nmol I-ol/l. An average body weight of 230 g and a blood volume of 14.5 ml was assumed. The molecular mass of I-ol is 240 g/mol. An oral bioavailability of 0.1% was used despite reasonable doubt about the finality of this amount.

Consequently, for 4 and 40 mg /kg the calculated blood concentration is 263 nmol/l and 2.63 µmol/l.

Thus, the concentration of I-ol in the cell experiments was about 15 times higher than in the animal experiment group with the highest dosage.

8) *"Figure 5: ALT/ AST values appear very low compared to literature, please check if the shown measurements are correct."*

We would like to take this opportunity to express our explicit thanks for this correct objection. In case of ALT and AST the labeling of the y-axis was chosen incorrectly. The enzymatic activities of these liver enzymes should be initially presented as µmoles/min/l instead of IU/l. This explains the low activity values, as conversion to IU/l involves multiplication by a factor of 60. The two diagrams a) and b) in figure 5 have been revised accordingly.

9) *"Figure 6: Is the total magnification 100x? Please provide pictures with a comparable background (white) correction. Also for histological evidence of steatosis/ fibrosis we advise to perform an Oil red O/ Sirius red staining which would be quantifiable."*

This is correct, the total magnification is x100, while the objective is x10. The total magnification has been inserted into the corresponding illustration legend instead of the objective magnification. The images have been corrected for a comparable background (white).

The staining with Sudan and Azan should allow the simultaneous computer assisted morphological evaluation of steatosis and fibrosis. The result of the semi-quantitative analysis is given in the S6 Table. The changes were incorporated in the manuscript.

10) *"Supplementary Table 6: The control value provided for TNF appears beneath those stated in other literature. In addition, the standard curve for the ELISA used for TNF evaluation appears to have limit of 100pg/µl. Please show the standard curve for this experiment."*

This is correct, the displayed values must be corrected by a factor of 10, S7 Table was updated accordingly. Enclosed is the corresponding standard curve.

**Figure 2: Calibration curve for determining of TNF-α serum level**

11) *"To add to the functional assays performed in this study it appears reasonable to provide some gene expression/ western blot data regarding the downstream effects of the drug on CYP2E1."*

Our defined goal was the development of a lead compound for the potential therapy of AFLD based on rational drug design. The insufficient data available at the time on important target proteins made this project considerably more difficult, so that we had to start with in silico calculations. If successful, the lead compound should be tested on animal models to determine only the clinically relevant parameters. If the results would prove to be statistically significant, we would consider a possible optimisation of this lead compound and possibly carry out further investigations, such as clarifying downstream target effects and off-target effects. To reach this point, however, very substantial resources had to be invested. After completion of this large project, we believe that further investigations on the intracellular effects of I-ol are indicated in separate projects.

## *"HepG2 cell line: Please provide further information regarding HepG2 E47 in a supplement, e.g. PCR/ WB showing the successful transfection."*

Both cell lines, HepG2 E47 and HepG2 C34, were kindly provided to us by Prof. Dr. Cederbaum (Icahn School of Medicine at Mount Sinai, NY) for research purposes in 2012. He first published the generation of these cells in subsequent publications: (a) Chen Q, Cederbaum AI. Cytotoxicity and Apoptosis Produced by Cytochrome P450 2E1 in Hep G2 Cells. Molecular Pharmacology. 1998;53(4):638-48. (b) Mari M, Cederbaum AI. CYP2E1 overexpression in HepG2 cells induces glutathione synthesis by transcriptional activation of gamma-glutamylcysteine synthetase. J Biol Chem. 2000;275(20):15563-71. Over the years, these publications have been the basis for numerous highly important studies on the intracellular effects of CYP2E1, especially the associated ROS production. At no time did we have any doubt about the authenticity of these cell lines. Furthermore, the increased ROS production in the HepG2 E47 cell line compared to the other two cell lines HepG2 and C34 was in line with the data published so far.

12) *"As a general remark, we understand the experimental setup of administering the drug daily alongside the LDC diet. Nevertheless, treating an organism while inducing ASH is hardly translatable to the human situation. Did you do perform any experiment where disease progression was ongoing for some time before the treatment was started? Could you speculate on how this would influence the efficacy of the treatment e.g. stop disease progression/ reverse steatosis … ?"*

In fact, the experimental design must be different if the disease of steatohepatitis has already manifested itself. This proof of concept is therefore still pending. Our approach can be understood as a preventive one, but this approach only partially reflects our considerations during the design of the trial. From the inhibition studies we know that I-ol competitively inhibits CYP2E1 and thus prevents ROS production. In addition, I-ol stabilizes the protein scaffold of CYP2E1 via a complex bond with heme iron. This experimentally proven knowledge is followed by considerations based on previous work on the stability of haemoproteins, CYP450 and CYP2E1. A descriptive summary can be found in the document "Pathophysiological Function of CYP2E1". Basically, we formulated the hypothesis that the in vivo stabilization of CYP2E1 stabilizes the redox system as a whole and that the reduced ROS formation can be attributed specifically to a functional elimination of CYP2E1 and not to other ROS-producing systems (respiratory chain, NADPH oxidase, xantine oxidoreductase). This "preventive" approach should reflect this causality. The therapy of an already manifested steatohepatitis cannot guarantee this because CYP2E1 is no longer mainly responsible for ROS formation and other or further pathophysiological processes are also relevant.

In a parallel project it was investigated whether I-ol is a possible drug candidate for the therapy of non-alcoholic steatohepatitis (NASH). In this case, no preventive approach was chosen, since the pathophysiology of ASH and NASH differ from each other despite common features. CYP2E1 is therefore only one possible drug target and the causality between CYP2E1 overexpression, ROS formation and steatohepatitis is less evident. The composition of the groups and the experimental procedure are shown in the following figure.

**Table 2:** **Group composition and time scale of the NASH (nonalcoholic steatohepatitis) study**

Compared to the ASH study, the results were less impressive. Surprisingly, however, the untreated animals did not show the full picture of steatohepatitis and possibly the treatment period of 4 weeks was also too short. If we were to choose a comparable experimental procedure for the therapy of ASH, the results could also be less impressive. However, before such a project is implemented, the knowledge of the pharmacokinetic properties of I-ol should be expanded.

**Reviewer 2**

**Major comments:**

13) *The title of the article as well as some parts of the paper that relate to ASH are imprecise. The authors present data to support the effect of their drugs in alcohol-induced liver injury and steatosis, but not steatohepatitis. None of the markers of liver inflammation were assessed. Most importantly, the model the author used is rather a model of early stages of ALD.*

We wanted to proof whether our active substances influence traditional liver markers like ALT, AST and Alkaline Phosphatase, which are accepted for clinical studies and liver inflammation as well, first. We know they give limited information concerning the state of a liver especially as “stand-alone” values.

Nevertheless, we believe that the used animal model reflects the early stages of steatohepatitis and not only steatosis, as there are signs of liver inflammation. Histological examinations clearly show the presence of necrotic areas and lymphocytic infiltrations in the liver slices of the disease group. These also show a significant increase in the serum content of TNF-α. For this reason, we have specified the terminology at appropriate points and used early stage alcoholic steatohepatitis.

In further studies, serum markers could be used, which are currently evaluated and might give more precise information about the state of a liver and were able to distinguish steatosis and steatohepatitis as this might be panels of gene transcription.

14) *One of my major concerns is why the authors chose to test the potentials drug in a preventive treatment dosing as opposed to using the drugs for reversing an established ALD. This would be more important information that is relevant to clinical practice. In my opinion, this is a major limitation of the present study.*

The pharmacological therapy of an alcoholic's liver that has already been fibrotically transformed would be a milestone in liver research. All development projects involving the therapy of fibrotic organs would have to be measured by this success. A causal relationship between CYP2E1 and fibrosis of the liver has not yet been explicitly investigated. It can be assumed that CYP2E1 plays an ever decreasing pharmacologically relevant role as the disease progresses via fibrosis and cirrhosis to the development of hepatocellular carcinoma (HCC). We wanted to take the first step and see whether others could follow. This first step is the demonstration that by competitively inhibiting CYP2E1, I-ol inhibits the formation of pathophysiologically relevant ROS and may prevent the development of ASH. These considerations led us to the conscious decision that for the time being we are not interested in a proof-of-concept study in which our drug candidates are used to cure ASH in advanced fibrotic stage or liver cirrhosis.

Nevertheless, in order to enable greater transparency in the discussion of the data, we have taken this objection into account by adding a content modification to the manuscript (see Discussion page lines 739-744 in "Revised Manuscript with Track Changes").

15) *What were the bodyweight changes during the treatment study?*

**Figure 3: Body weight dynamics**

For a more comprehensive overview, the data are not shown with the corresponding error bars. A significant difference between the groups could not be determined.

16) *What was the final liver weight and liver-to-body weight ratio?*

**Figure 4: Final liver weight**

A significant difference between the groups could not be determined.

**Figure 5: Final body weight**

A significant difference between the groups could not be determined.

**Figure 6: Ratio between final liver weight and final body weight**

A significant difference could only be found between the disease group and the control group.

17) *Was diet intake measured and caloric intake the same? These data should be included in the manuscript.*

The food intake was measured. This was lower in all groups whose animals were fed with ethanol than in the control group. There was no significant difference between the groups fed with ethanol. The calorie value of both the control and ethanol diets was the same: the Lieber-DeCarli liquid alcohol diet contained 35% calories from fat, 11% from carbohydrates, 18% from protein and 36% from ethanol. Ethanol was replaced by the isocaloric amount of maltodextrin in the Lieber-DeCarli liquid control diet. A corresponding text has been added in lines 272 - 274 of the document "Revised Manuscript with Track Changes".

***Table 3: High-fat Lieber-Decarli diet consumption, g/day per 1 rat***

18) *Figures are not easy to understand and follow. To make figures more intuitive, the authors can label graphs and images with more details (e.g., parameters assessed, use graph titles, treatment, legends, etc.)*

If the figures and tables do not meet the standard criteria of PLOS One, they will of course be revised. In the current version of the submitted manuscript these seem to be fulfilled.

**Figure 7: Commented reference figure on the PLOS One website**

**Minor comments:**

19) *Any* abbreviation, such as ASH, should be avoided in the title to improve understanding of the general audience. But as noted above, ASH should not even be mentioned as a major focus of the paper.

Abbreviations were removed from the manuscript at the appropriate place. As mentioned earlier in our opinion, the animal model should adequately represent the early ASH stage. To provide more clarity of content, the manuscript was corrected accordingly (see Discussion page lines 739-744 in "Revised Manuscript with Track Changes").

20) *Replace “steatosis hepatitis” with appropriate terms – page 3, twice.*

Throughout the manuscript the term "steatosis hepatitis" is not used, but "steatosis hepatis". To our knowledge, an alternative formulation which reflects the pathophysiological state of the liver in the same way is not currently common.

21) *In the introduction, liver enzymes are not spelled out to introduce the abbreviation. Also, the abbreviations are not the most commonly used ones in the liver literature.*

Liver enzymes are mentioned in the introduction of the manuscript on page 5 with the sentence "The liver enzymes ASAT, ALAT, γ-GT and AP had lost catalytic activities in the treatment groups." Since the abbreviations AST and ALT are used in the rest of the manuscript, we have also used them for the introduction.

22) *Actually, at multiple places of the manuscript several abbreviations are not introduced. Too many abbreviations make the manuscript less well readable, and especially if the abbreviations are quite specific to the field, e.g. chemistry vs. hepatology.*

This work applies the results from the field of rational drug design to a clinical problem, namely the therapy of alcoholic steatohepatitis. Before preparing the manuscript, we had to decide whether to split it into two topics and publish them separately: (a) rational drug design with the fields of computational chemistry, organic and analytical chemistry and enzyme kinetics (b) hepatology. Since our team had experts from all of the above mentioned fields and since the proof-of-concept in animal models was always in mind at the beginning of the project, we decided to publish a manuscript containing all studies and results. In particular, readers from the field of hepatology will check the findings for relevance within their own discipline. For this reason, the section on drug development takes up a quantitatively much smaller part, which is why certain aspects cannot be adequately explained. This also includes abbreviations that are common for readers from the field of drug development. In order to provide access to the contents of the manuscript for all interested readers, very specific terms and their abbreviations were intentionally omitted in the sections Abstract, Introduction and Discussion.

23) *Many references throughout the manuscript were not inserted; see words ‘Error” in the text.*

Four internal links did not work in the PDF document after it was automatically created from the Word document. The reason for this error message was missing links of the supplement tables and files in the text. These are now fixed.

24) *Listing % values in figure legend is not very informative and should be omitted.*

The illustration of each individual measured value of each animal within each group creates maximum transparency. Besides the question whether there were statistically significant changes between the disease group and the two control groups, the main question was to be answered: Are there significant differences between the six experimental substance groups and the control group with UDCA compared to the disease group? If so, how big are these differences? Many common laboratory values in clinical routine do not show massive changes, especially in chronic processes. In addition, drug therapy successes in these disorders often show slight to moderate changes in the clinical, especially the laboratory picture. From our point of view, the reporting of the differences in percentage values is very well suited for quantifying the changes.
